# Antiviral Activity of *Origanum vulgare* ssp. *hirtum* Essential Oil-Loaded Polymeric Micelles

**DOI:** 10.3390/biomedicines13102417

**Published:** 2025-10-02

**Authors:** Neli Vilhelmova-Ilieva, Ivan Iliev, Katya Kamenova, Georgy Grancharov, Krasimir Rusanov, Ivan Atanassov, Petar D. Petrov

**Affiliations:** 1 The Stephan Angeloff Institute of Microbiology, Bulgarian Academy of Sciences, Acad. G. Bonchev Str., 26, 1113 Sofia, Bulgaria; nelivili@gmail.com; 2 Institute of Experimental Morphology, Pathology and Anthropology with Museum, Bulgarian Academy of Sciences, Acad. G. Bonchev Str., 25, 1113 Sofia, Bulgaria; taparsky@abv.bg; 3 Institute of Polymers, Bulgarian Academy of Sciences, Acad. G. Bonchev Str., Bl. 103A, 1113 Sofia, Bulgaria; kkamenova@polymer.bas.bg (K.K.); granchar@polymer.bas.bg (G.G.); 4 Centre of Competence “Sustainable Utilization of Bio-Resources and Waste of Medicinal and Aromatic Plants for Innovative Bioactive Products” (CoC BioResources), 1000 Sofia, Bulgaria; krusanov@abv.bg (K.R.); ivan_atanassov@abv.bg (I.A.); 5 Department of Agrobiotechnology, AgroBioInstitute, Agricultural Academy, 1164 Sofia, Bulgaria

**Keywords:** nanocarriers, oregano essential oil, herpes simplex virus type 1, human coronavirus, feline calicivirus, virucidal activity

## Abstract

**Background**: Encapsulating essential oils in polymer-based nanocarriers can improve their stability, solubility, and bioavailability, while maintaining the biological activity of the oil’s active ingredients. In this contribution, we investigated the antiviral activity of Oregano Essential Oil (OEO) in its pure form and encapsulated into nanosized polymeric micelles, based on a poly(ethylene oxide)-block-poly(ε-caprolactone) diblock copolymer. **Methods**: The effect of encapsulation was evaluated using three structurally different viruses: herpes simplex virus type 1 (HSV-1) (DNA—enveloped virus), human coronavirus (HCoV OC-43) (RNA—enveloped virus), and feline calicivirus (FCV) (RNA—naked virus). The effect on the viral replicative cycle was determined using the cytopathic effect inhibition (CPE) test. Inhibition of the viral adsorption step, virucidal activity, and protective effect on healthy cells were assessed using the final dilution method and were determined as Δlg compared to the untreated viral control. **Results**: In both studied forms (pure and nanoformulated), OEO had no significant effect on viral replication. In the remaining antiviral experiments, the oil embedded into nanocarriers showed a slightly stronger effect than the pure oil. When the oil was directly applied to extracellular virions, viral titers were significantly reduced for all three viruses, with the effect being strongest for HSV-1 and FCV (Δlg = 3.5). A distinct effect was also observed on the viral adsorption stage, with the effect being most significant for HSV-1 (Δlg = 3.0). **Conclusions**: Pretreatment of healthy cells with the nanoformulated OEO significantly protected them from viral infection, with the greatest reduction in viral titer for HCoV OC-43.

## 1. Introduction

More than 20% of mortality in the human population is due to the development of various infectious diseases, one third of which are caused by viruses [1]. The interaction between the virus and the cell in which it multiplies is very complex, because for obtaining new viral particles, different viruses follow different mechanisms, using the synthetic apparatus of the host cell. Therefore, it is difficult to develop specific antiviral therapeutics that inhibit viral replication but do not affect the structures of the cell. The chemotherapeutics developed so far exhibit numerous side effects, and have the disadvantage that viruses quickly form resistant mutants against the applied therapeutic. This leads to failure of therapy. Therefore, there is a need to develop new antiviral therapeutics that have fewer side effects and lead to a more difficult selection of resistant mutants.

For years, scientists have turned their attention to the study of various natural products. Many of them have shown a positive influence on various disease states, including numerous infectious diseases. Recently, more data has been accumulating on the antiviral activity of various types of essential oils. Most antiviral studies have focused on enveloped viruses, as essential oils interact with the viral envelope structures of these viruses. For example, in human herpes viruses (HSV-1 and HSV-2), essential oils from Star Anise, Australian Tea Tree, Oregano, and Eucalyptus caesia have been shown to exhibit antiviral activity [2,3]. Cymbopogon nardus essential oil inhibits HIV-1 reverse transcriptase activity [4]. It has been suggested that potential inhibitors of COVID-19 are essential oil-derived components such as 1,8-cineole [5] and isothymol [6]. Antiviral effects of essential oils and their components have also been observed on respiratory syncytial virus [7], bovine viral diarrhea virus [8], yellow fever virus [9], Zika virus [10], and caprine alphaherpesvirus [11]. Non-enveloped viruses have received relatively less attention because they have fewer active sites that essential oil components can target. However, there is also evidence of inhibitory effects against non-enveloped viruses [12]. Essential oils from Osmunda regalis, Eucalyptus bicostata, and Dysphania ambrosioides have shown high antiviral activity against non-enveloped Coxsackie virus [13,14]. Oregano essential oil inhibits human rotavirus and murine norovirus [8,15]. Oregano essential oil can target different phases of the viral life cycle, such as extracellular virus, viral adsorption, internalization, or genome replication [12]. The main biological activities, including antiviral activity, of oregano oil are attributed to its main components, carvacrol and thymol. Carvacrol and thymol have been shown to exhibit antiviral activity against a number of viruses [8,16,17,18], especially when pretreated. Studies by two teams have demonstrated the anti-HIV and anti-SIV effects of oregano essential oil, carvacrol, and thymol, mainly through their direct interaction with the structure of the viral capsule [19,20]. Other studies have confirmed that oregano essential oil and carvacrol only have a direct killing effect against pseudorabies virus (PRV) and limit intracellular virus proliferation but do not inhibit PRV invasion into BHK-21 cells [21]. Oregano oil also inactivates murine norovirus within 1 h by acting directly on the viral capsid and subsequently affecting RNA [15]. Numerous studies have shown that oregano oil can directly kill lipid-encapsulated viruses [22]. Studies have shown that carvacrol and thymol block viral fusion with cells without disrupting other stages of the viral life cycle. Changes in the density of viral particles have been found, suggesting that cholesterol depletion from the HIV-1 envelope membrane occurs. Cholesterol is an important component of the viral bilayer membrane, required for viral stability and efficient fusion with the cell membrane, which results in reduced viral entry [23,24]. The dependence on cholesterol for viral entry has also been demonstrated for other enveloped viruses, such as HCV and influenza [25,26,27]. In silico studies revealed that carvacrol and thymol may show moderate activity as G-protein coupled receptor ligands, ion channel modulators, nuclear receptor ligands, and protease, kinase, and enzyme inhibitors [28].

However, the components contained in OEO have extremely low solubility in water, which is the natural solvent in all living organisms. This makes the application of OEO in practice difficult and compromises its effects. To avoid this drawback of the oil, various methods are being developed for its introduction into nanocarriers [29,30,31,32]. For example, some of its biological activities, such as antibacterial, antifungal, or antioxidant, have been improved by encapsulation in chitosan–alginate nanoparticles [33], chitosan nanoparticles [34], polyvinyl alcohol–chitosan nanoparticles [35], and poly(ε-caprolactone) nanoparticles [36]. There have also been positive results in the study of antiviral activity. When combining a nanogel (based on the natural polymers chitosan and albumin) with oregano oil, MDBK cells were effectively protected from betacoronavirus 1 viral infection [37].

Furthermore, the incorporation of essential oils into polymer-based nanocarriers, such as micelles, nanocapsules, nanogels, etc., reduces the volatility and improves the stability of the active ingredients of the oils, which is a key issue for preserving their biological activity [38,39,40].

Polymeric micelles (PMs) are particles with a size usually between 10 and 100 nm that, in recent decades, have been widely studied for application in medicine as carriers of drugs and diagnostic tools. PMs can be formed by self-association of amphiphilic block copolymers, containing hydrophilic and hydrophobic segments, in an aqueous environment. Most often, the obtained PMs possess a spherical structure of the “core–shell” type. This architecture, characterized by an inner hydrophobic core and an outer hydrophilic shell, determines the ability of micelles to encapsulate hydrophobic molecules, ensuring their aqueous solubility and simultaneous protection from the environment [41]. Essential oils are liquids that can be easily encapsulated in the hydrophobic micelle core, thus providing possibilities for increased stability and circulation period in the body, and also controlled and/or targeted release into target cells. The polymers used for preparing PMs can be of natural or synthetic origin. Among the most commonly used synthetic polymers approved for pharmaceutical use are poly(ethylene oxide) (PEO), poly(lactic acid) (PLA), poly(glycolic acid) (PGA), poly(ε-caprolactone) (PCL), and poly(vinyl alcohol) (PVA) [42,43].

It is known that amphiphilic block (or graft) copolymers composed of a hydrophilic PEO block and a hydrophobic, biodegradable PCL block (PEO-PCL) self-assemble in aqueous media into core–shell micelles. The PCL core serves as a reservoir of poorly water-soluble bioactive compounds (essential oils, anticancer agents, etc.), while the PEO shell provides water solubility, steric stabilization, and limited interactions with plasma proteins, named the “stealth” effect. PEO-PCL systems are known for their low toxicity and high loading capacity, making them attractive candidates for pharmaceutical and biomedical applications [44,45,46].

The present work aims at developing oregano essential oil-loaded polymeric micellar systems with antiviral activity. Firstly, polymeric micelles from a poly(ethylene oxide)-block-poly(ε-caprolactone) diblock copolymer (PEO_113_-*b*-PCL_29_) were prepared by the solvent evaporation method, and then OEO was incorporated into nanocarriers via hydrophobic interaction. The nanosystems were characterized by dynamic and electrophoretic light scattering (DLS and ELS), UV–Vis spectroscopy, and atomic force microscopy (AFM). Next, a comparative study of the antiviral activity of nanoformulated and pure OEO was carried out by using three viruses from different virus families. The selected viruses have different structures as follows: (i) herpes simplex virus type 1, which contains DNA wrapped in an icosahedral capsid covered by an envelope, composed of a lipid bilayer containing many proteins (supercapsid) [47]; (ii) coronavirus, whose genome consists of a single-stranded positive RNA wrapped in a capsid and, like the herpes virus, has a lipoprotein envelope [48]; (iii) feline calicivirus, whose genome is single-stranded RNA wrapped only by a capsid [49]. This virus does not contain a supercapsid and is one of the so-called naked viruses.

## 2. Materials and Methods

### 2.1. Materials

The diblock copolymer poly(ethylene oxide)-block-poly(ε-caprolactone) (PEO_113_-*b*-PCL_29_, Mn = 8310 g/mol) was synthesized as described elsewhere [50]. The synthesis procedure and characterization data are shown in the Supplementary Materials. Ethanol (99.5%), acetone (99%), and phosphate buffer pH = 7 were purchased from Sigma-Aldrich via FOT, Sofia, Bulgaria, and used as received. Vegetatively propagated plants of line 149/4/iv3 of *O. vulgare* ssp. *hirtum*, grown in the experimental field of the AgroBioInstitute in nearby Kostinbrod, Bulgaria, were used in the study. Line 149/4/iv3 was selected in 2019 from a wild population in the Rhodope Mountains, Bulgaria [51]. The distillation of OEO was carried out as described elsewhere [52]. The chemical composition of OEO was determined by GC–MS analysis (see Supplementary Materials).

### 2.2. Preparation of PEO-b-PCL Block Copolymer Micelles

The nanosized aggregates were obtained by the solvent evaporation method. Briefly, 50 mg of polymer was dissolved in 4 mL of acetone. The solution was then added dropwise to 10 mL of deionized water at room temperature under continuous stirring (IKA-Werke GmbH & Co. KG, Staufen, Germany). After 30 min, the organic solvent was evaporated using a rotary vacuum evaporator. The concentration of the resulting colloidal aqueous dispersion was 5 mg/mL.

### 2.3. Preparation of OEO-Loaded PEO-b-PCL Block Copolymer Micelles

Oregano essential oil was loaded into the polymer nanocarriers at OEO/polymer mass ratios of 1:5, 1:2.5, and 1:1, according to the following procedure: An ethanol solution of OEO (10 mg/mL) was added to 5 mL of the previously prepared micellar dispersion (5 mg/mL) in the following volumes: 0.5 mL (mass ratio 1:5), 1.25 mL (1:2.5), and 2.5 mL (1:1). The organic solution was added dropwise to the aqueous colloidal solution under continuous stirring. After 30 min, the organic phase was removed under vacuum using a rotary evaporator. The resulting colloidal aqueous dispersions had a polymer concentration of 5 mg/mL and OEO concentrations of 1 mg/mL, 2.5 mg/mL, and 5 mg/mL, respectively. The major bioactive compounds of OEO, carvacrol and thymol, were quantified by GC–MS before and after incorporation into polymeric micelles. The data confirmed that the encapsulation procedure did not alter the composition of the essential oil [52].

### 2.4. In Vitro Release of OEO

In vitro release of OEO from the polymeric micelles was studied in a buffer medium (pH 7) at 37 °C using a dialysis method. Briefly, 4 mL of OEO-loaded PCL-PEO micelles were placed into a dialysis membrane (8000 MWCO, Spectrum Labs, San Francisco, CA, USA) and then submerged in 80 mL of release buffer medium, pre-heated to 37 °C. At certain time intervals, 2 mL samples were taken, and the same volume of fresh medium was added. The concentration of the released OEO was determined by UV–VIS spectrophotometry at λ = 273 nm, using a calibration curve in phosphate buffer with 1% ethanol (r > 0.9999) (Supplementary Figure S3).

### 2.5. Analysis

Quantitative analysis of the volatile compounds of oregano essential oil was performed by GC–MS using an Agilent 8890 GC system (full details in Supplementary Materials). The hydrodynamic diameters (D_h_, z-average) of blank and OEO-loaded polymeric micelles were measured at 25 °C using a Zetasizer NanoBrook 90Plus PALS (Holtsville, NY, USA) equipped with a 35 mW red diode laser (λ = 640 nm) at a scattering angle of 90°. The ζ-potential measurements were conducted at a scattering angle of 15° and the ζ-potential was calculated from the obtained electrophoretic mobility at 25 °C. The turbidity of the colloidal dispersions was evaluated by measuring light transmittance using a UV–Vis spectrophotometer (Thermo Scientific, Waltham, MA, USA). Measurements were performed in quartz cuvettes with a 1 cm path length. Transmittance was recorded at 500 nm. AFM images were taken using a Bruker Dimension Icon microscope (Bruker Corporation, Karlsruhe, Germany) in ScanAsyst mode in air. Samples were prepared by spin-coating a small volume of the OEO-loaded micellar dispersion (2 μL) at 2000 rpm onto a glass slide, followed by air-drying at room temperature. The resulting height and phase images were processed and analyzed using Bruker’s NanoScope Analysis software V1.50R2SR1.

### 2.6. Host Cell Lines

Madin–Darbey bovine kidney (MDBK) cells, Crandell–Rees Feline Kidney (CRFK) cells, and Vero E6 (isolated from the kidney of an African green monkey) cells were provided by the National Bank for Industrial Microorganisms and Cell Cultures, Sofia, Bulgaria. Cells were grown in DMEM growth medium (Gibco, Grand Island, NY, USA) containing 10% fetal bovine serum (Gibco, Grand Island, NY, USA), supplemented with 10 mM HEPES buffer (AppliChem GmbH, Darmstadt, Germany) and antibiotics (penicillin 100 IU/mL, streptomycin 100 μg/mL). Incubation was performed in an incubator (HERA cell 150, Heraeus, Hanau, Germany) at 37 °C and a 5% CO_2_ atmosphere.

### 2.7. Viruses

Human Coronavirus OC-43 (HCoV-OC43, ATCC: VR-1558) (ATCC, Manassas, VA, USA) strain was propagated in Vero E6 cells in DMEM medium supplemented with 2% fetal bovine serum, 100 U/mL penicillin, and 100 μg/mL streptomycin. Cells were lysed for 5 days after infection by 2 freeze and thaw cycles, and the virus was titrated according to the Reed and Muench formula [53]. Virus and mock aliquots were stored at −80 °C. The infectious titer of the stock virus was 10^6.5^ CCID_50_/mL.

Herpes simplex virus type 1, Victoria strain (HSV-1), was received from Prof. S. Dundarov, National Center of Infectious and Parasitic Diseases, Sofia. The virus was replicated in a confluent monolayer of MDBK cells.

Feline calicivirus (FCV) (from the collection of the Institute of Microbiology, Bulgarian Academy of Sciences) is propagated in cells of the CRFK cell line. In the case of HSV and FCV, the infected cells are incubated in the presence of Dulbecco’s modified Eagle’s medium (DMEM) Gibco BRL, Paisley, Scotland, UK, plus 0.5% fetal bovine serum (Gibco BRL, Scotland, UK) and antibiotics (penicillin 100 IU/mL, streptomycin 100 μg/mL). After incubation at 37 °C in a 5% CO_2_ incubator, the viral yield was frozen at −80 °C. The infectious viral titer of the initial virus stock was 10^8.5^ CCID_50_/mL for HSV and 10^7.25^ CCID_50_/mL for FCV.

### 2.8. Reference Compound

Remdesivir (GS-5734, RDV, REM, Veklury^®^) (Gilead Sciences, Carrigtwohill, Ireland) was initially dissolved in double-distilled water to a concentration of 150 mg/mL and then diluted in RPMI nutrient medium to the required concentrations.

Acyclovir {ACV, [9-(2-hydroxyethoxymethyl)-guanine]} was kindly provided by the Deutsches Kresforschung Zentrum, Heidelberg, Germany, with a stock concentration of 3 mM solution in DMSO. Then, falling dilutions were made in DMEM medium to the required concentration.

### 2.9. In Vitro Safety Testing

Mouse embryonic fibroblasts (BALB/3T3 clone A31, ATCC: CCL-163™) were used to perform the safety test. The cells were obtained from the American Type Cultures Collection (ATCC, Manassas, VA, USA). The safety test was performed by OECD Guidelines for the Testing of Chemicals, Section 4, Test No. 432 [54]. Cells were plated at 1 × 10^4^ cells/well in 96-well microplates. After 24 h of incubation, the test substances were added at various concentrations (from 10 to 2500 μg/mL). In the phototoxicity test, the plates were irradiated with a dose of 2.4 J/cm^2^ using a solar light simulator Helios-iO (SERIC Ltd., Tokyo, Japan). Cytotoxicity is presented as % relative to the negative control (untreated cells). The 50% cytotoxic concentration (CC_50_) was defined as the concentration of the extract that reduced cell viability by 50% compared to untreated controls [51]. The CC_50_ values were used to calculate the Photo-Irritation Factor (PIF), according to the formula:

(1) $$PIF=\frac{{\mathrm{CC}}_{50}\left(non-irradiated\right)}{{\mathrm{CC}}_{50}\left(irradiated\right)}.$$

### 2.10. Cytotoxicity Assay

A confluent monolayer cell culture in 96-well plates (Costar^®^, Corning Inc., Kennebunk, ME, USA) was treated with 0.1 mL/well containing support medium that did not contain or contained decreasing concentrations of the samples tested. Cells are incubated under the characteristic conditions under which subsequent virus experiments will be performed, at 33 °C and 5% CO_2_ for 5 days (for HCT-8) and 2 days at 37 °C and 5% CO_2_ for MDBK and CRFK cells. After the given period of time, the tested extracts were removed. The cells were washed and incubated with neutral red (NR) dye at 37 °C for 3 h. The CC_50_ and the maximally tolerated concentration (MTC) of the tested samples, at which they do not affect the morphology of the cell monolayer, were also determined. Each sample was tested in triplicate with four wells per replicate.

### 2.11. Determination of Infectious Viral Titers

In 96-well plates, a monolayer of HCT-8, MDBK, or CRFK cells was infected with 0.1 mL of virus suspension at tenfold decreasing dilutions [55]. After the virus adsorption period, the unabsorbed virus was removed, and the DMEM medium was added. This was followed by incubation at 37 °C and 5% CO_2_ in a HERA cell 150 CO_2_ incubator (Radobio Scientific Co., Ltd., Shanghai, China) for 48 h (for HSV-1 and FCV) and 120 h (for HCoV-OC43). Cells infected with the maximum concentration of the virus and demonstrating the maximum cytopathic effect were used as controls. The resulting cytopathic effect (CPE) was monitored by microscopic observation of the cell monolayer and confirmed by neutral red uptake assay (Borenfreund and Puerner, 1985 [51]).

### 2.12. Antiviral Activity Assay

The cytopathic effect inhibition (CPE) test was used to determine the antiviral activity of propolis extracts [55]. A confluent cell monolayer in 96-well plates was infected with 100 cell culture infectious doses of 50% (CCID_50_) in 0.1 mL. After 2 h of adsorption at 33 °C (for HCoV OC-43) and 1 h of adsorption at 37 °C (for HSV-1 and FCV), unattached virus was removed and the tested sample was added at different concentrations and the cells were incubated for 5 days at 33 °C (for HCoV OC-43) or 2 days at 37 °C (for HSV-1 and FCV) and in the presence of 5% CO_2_. The cytopathic effect was determined using a neutral red uptake assay and the percentage of CPE inhibition for each test sample concentration was calculated using the following formula:

(2) $$CPE(\%)=\frac{OD\;test\;sample-OD\;virus\;control}{OD\;toxicity\;control-OD\;virus\;control}\times 100$$

where OD test sample is the mean of the ODs of the wells inoculated with virus and treated with the extract at the corresponding concentration, ODs virus control is the mean of the ODs of the virus control wells (no compound in the medium), and OD control for toxicity is the mean of the ODs of the wells not inoculated with virus but treated with the corresponding concentration of the extract.

### 2.13. Effect on Viral Adsorption

Twenty-four well plates containing a monolayer of HCT-8, MDBK, or CRFK cells were pre-chilled to 4 °C and inoculated with 10^4^ CCID_50_ of HCoV OC-43, HSV-1, or FCV, respectively. Along with the virus, the monolayer was also treated with the tested samples in their MTC and incubated at 4 °C for the time of virus adsorption. At different time intervals different for the types of virus (15, 30, 45, and 60 min for HSV-1 and FCV or 15, 30, 60, 90, and 120 min for HCoV OC-43), the virus and the tested sample were removed, the cells were washed with PBS, then the cells were covered with maintenance medium and incubated at 37 °C (HSV-1 and FCV) or at 33 °C (for HCoV OC-43) in the presence of 5% CO_2_ for 24 h. After freezing and thawing three times, the infectious viral titer of each sample was determined, compared to the viral titer of the control for the given time interval, and Δlgs were determined. Each sample was prepared in quadruplicate.

### 2.14. Virucidal Assay

Samples with a total volume of 1 mL containing virus (10^5^ CCID_50_) and propolis extract at its maximally tolerated concentration (MTC) in a 1:1 ratio were prepared. A sample containing untreated virus diluted 1:1 with DMEM medium was incubated in parallel. The control and experimental samples were incubated at room temperature for different time intervals (15, 30, 60, 90, and 120 min). Then, by the endpoint dilution method [51], the contents of residual infectious virus in each sample and Δlgs compared to untreated controls were determined.

### 2.15. Pre-Treatment of Healthy Cells

A monolayer of MDBK, CRFK, or HCT-8 cells previously grown in 24-well cell culture plates (CELLSTAR, Bishop’s Stortford, UK) was treated with the tested sample in their MTC. The samples were incubated for different time intervals of 15, 30, 60, 90, and 120 min at 37 °C. After the given time interval, the samples were removed, and the cells were washed with PBS and inoculated with the respective virus strain (10^4^ CCID_50_ in 1 mL/well). After 120 min (for HCoV OC-43) and 60 min (for HSV-1 and FCV) of adsorption, unadsorbed virus was removed, and the cells were covered with support medium. Samples were incubated at 33 °C (for HCoV OC-43) and 37 °C (HSV-1 and FCV) in the presence of 5% CO_2_ for 24 h. This was followed by triplicate freezing and thawing of samples, and the determination of infectious virus titers. Δlg compared to the viral titer of the control (untreated with sample) for the given time interval was determined. Each sample for each time interval studied was prepared in four replicates.

### 2.16. Statistical Analysis

Data on cytotoxicity and antiviral effects were analyzed statistically. The values of CC_50_ were presented as means ± SD. The differences’ significance between the cytotoxicity values of the samples tested and the reference substances, as well as between the effects of the test products on the viral replication, was determined by Student’s *t*-test; *p*-values of <0.05 were considered significant. The final data sets were analyzed with the Graph Pad Prism 4 software (San Diego, CA, USA).

## 3. Results

### 3.1. Preparation and Characterization of PEO-b-PCL Block Copolymer Micelles

**Figure 1:**
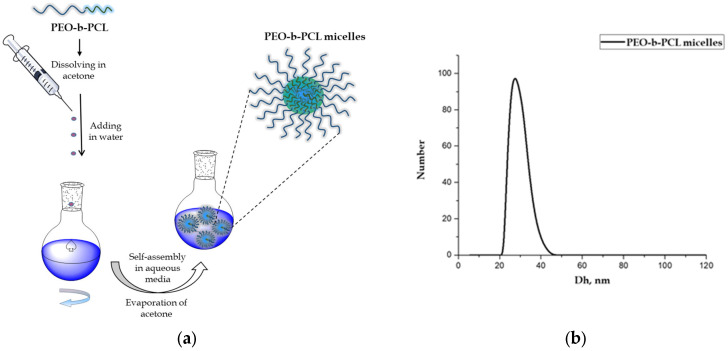
(**a**) Schematic illustration of the preparation of PEO-*b*-PCL micelles and (**b**) particle size distribution of blank PEO-*b*-PCL micelles.

Micellar nanocarriers from an amphiphilic diblock copolymer, composed of poly(ethylene oxide) and poly(ε-caprolactone) (PEO_113_-*b*-PCL_29_), were formed by self-assembly in aqueous media. The preparation procedure is illustrated in Figure 1a. The method involves dissolution of the copolymer in acetone, which is a good solvent for both blocks (PEO and PCL), followed by slow addition of the organic phase into water. Next, the organic solvent was evaporated under vacuum, resulting in a stable colloid solution containing micelles with a hydrophobic, biodegradable PCL core and a hydrated PEO shell (5 mg/mL). The as-obtained system was characterized by dynamic and electrophoretic light scattering. The results showed that the particles have an average hydrodynamic diameter (D_h_) of 27 nm (Figure 1b) and a negative zeta potential of approximately −8 mV.

### 3.2. Preparation and Characterization of OEO-Loaded PEO-b-PCL Block Copolymer Micelles

**Figure 2:**
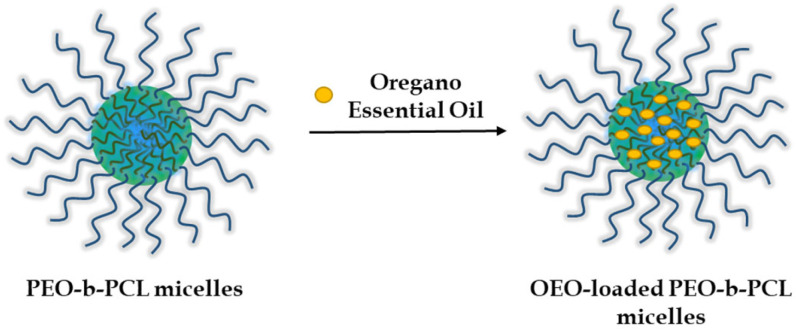
Schematic representation of the preparation of OEO-loaded PEO-*b*-PCL micelles.

In the next step, OEO (dissolved in ethanol) was embedded into the hydrophobic core of the micelle through hydrophobic interactions at three different OEO/polymer mass ratios—1:5, 1:2.5, and 1:1 (Figure 2).

**Figure 3:**
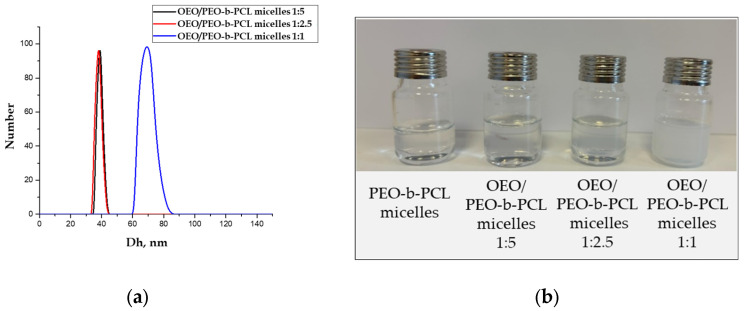
(**a**) Particle size distribution of OEO-loaded PEO-*b*-PCL micelles at OEO/polymer mass ratios of 1:5, 1:2.5, and 1:1, and (**b**) photographs of the corresponding colloid solutions.

The physicochemical characteristics of OEO-loaded PMs were investigated by dynamic and electrophoretic light scattering and turbidity measurements. The results are presented in Table 1. DLS measurements showed that the particle size of the loaded micelles increased as compared to the blank PMs (Figure 3a). The D_h_ of the particles loaded with oregano oil at 1:5 and 1:2.5 OEO/polymer mass ratio was approximately 39 nm. These two colloid solutions were less transparent (slightly opalescent) than the non-loaded micelles. Further increase in OEO amount (1:1 OEO/PEO-*b*-PCL mass ratio) led to a notable increase in the particle size (D_h_ = 69 nm) and the solution became more turbid (Figure 3b, Table 1).

**Figure 4:**
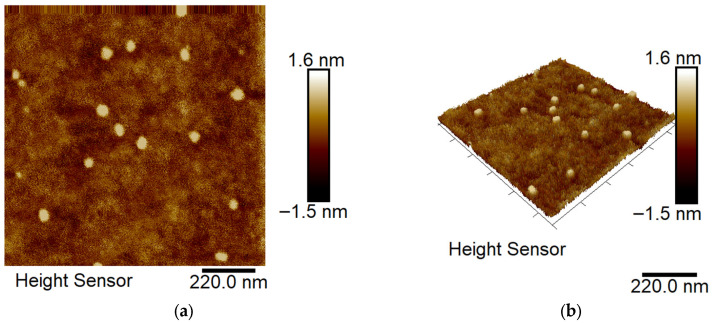
Atomic force microscopy 2D (**a**) and 3D (**b**) height images of OEO-loaded PEO-*b*-PCL micelles at mass ratio OEO/polymer 1:5.

Atomic force microscopy was performed to evaluate the surface morphology of OEO-loaded nanocarriers at an OEO/PEO-*b*-PCL micelles mass ratio of 1:5. The AFM images (Figure 4a,b) revealed that the dominant population of particles possesses a spherical shape with nanoscale dimensions. The particle size observed by AFM was 32 ± 6 nm, which corresponds well to that determined by DLS measurements.

**Figure 5:**
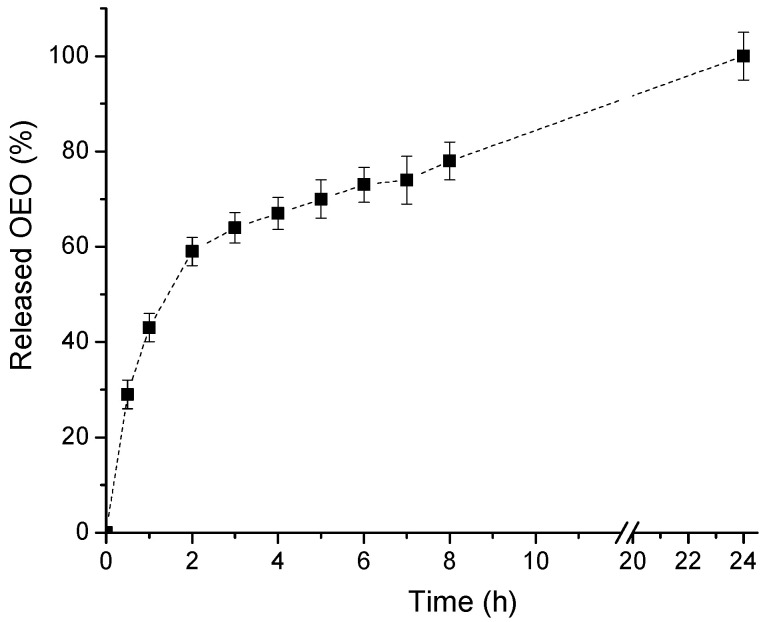
In vitro release of oregano essential oil in phosphate buffer (pH 7).

The release of OEO from PEO-*b*-PCL micelles (polymer/OEO mass ratio 5:1) in a phosphate buffer (pH 7) was assessed by using the dialysis method. As evident from Figure 5, a biphasic release profile was observed. A fast drug release of OEO (59%) within the first two hours was followed by a sustained release of the encapsulated essential oil for 24 h.

### 3.3. Biological Assessment

#### 3.3.1. Cytotoxicity

**Figure 6:**
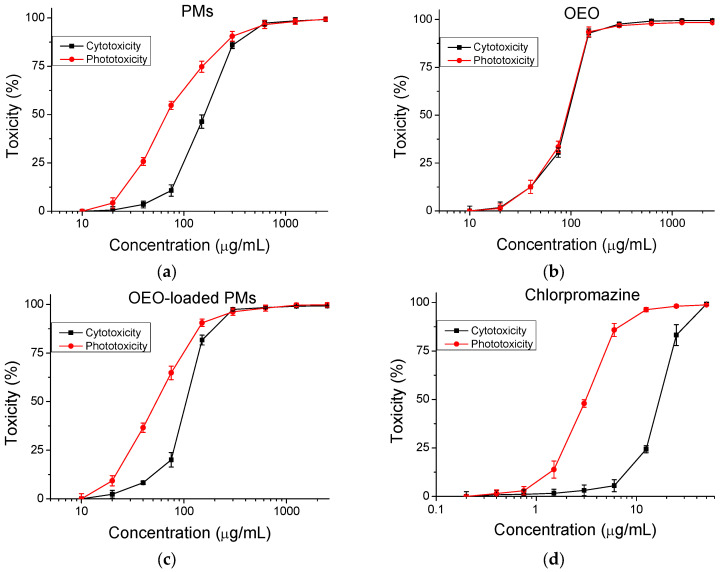
Cytotoxicity/phototoxicity of compounds in BALB/3T3 cells. (**a**) Polymer micelles (PMs), (**b**) oregano oil (OEO), (**c**) OEO-loaded polymer micelles, (**d**) Chlorpromazine (positive control). Values are means ± SD from three independent experiments, *n* = 6.

The safety of the tested products (cytotoxicity/phototoxicity) was assessed and compared to a negative control. Dose–response dependence was observed for all compounds, as shown in Figure 6. The CC_50_ values (50% cytotoxic concentration) were calculated and are presented in Table 2. The PIF value indicates the probability for the tested substance to cause a phototoxic effect (PIF < 2 not phototoxic; 2 ≤ PIF < 5 probable phototoxicity, PIF ≥ 5 phototoxic). The phototoxic drug Chlorpromazine was used as a positive control. The calculated PIF for the tested combination (copolymer and OEO) is <2, which indicates that the OEO-loaded polymer micelles are photosafe.

To correctly conduct the antiviral experiments, it was necessary to exclude any side effects of the toxicity of the tested products. For this purpose, the cytotoxicity of OEO, PEO-*b*-PCL micelles, and OEO-loaded PEO-*b*-PCL micelles on three cell lines (MDBK, Vero E6, CRFK) in which the replication of the three types of viruses under study takes place was investigated. In fact, the highest cytotoxicity against the three cell lines was shown by the pure OEO. The results were similar to the tested reference substances (acyclovir and remdesivir). The lowest cytotoxicity was demonstrated by the PEO-*b*-PCL micelles—about two times lower cytotoxicity than the reference substances. The OEO-loaded PEO-*b*-PCL micelles showed average cytotoxicity values. Based on the results obtained, it can be concluded that the inclusion of OEO in the nanocarrier leads to lower overall cytotoxicity compared to the pure OEO. Concerning the cytotoxicity of the tested samples with respect to the type of cells, it is seen that the lowest toxicity is found in MDBK cells. Similar CC_50_ values, but with slightly higher cytotoxicity, are observed in the CRFK cell line. The highest cytotoxicity is reported in Vero E6 cells. The results obtained were expected because the cytotoxicity determination for each cell line was performed at the same time as the antiviral experiments. For MDBK and CRFK cell lines, the effect was determined at 48 h, and for Vero E6 cells, it was performed at 120 h, and, logically, then the cytotoxicity is higher (Table 3).

#### 3.3.2. Antiviral Activity

**Figure 7:**
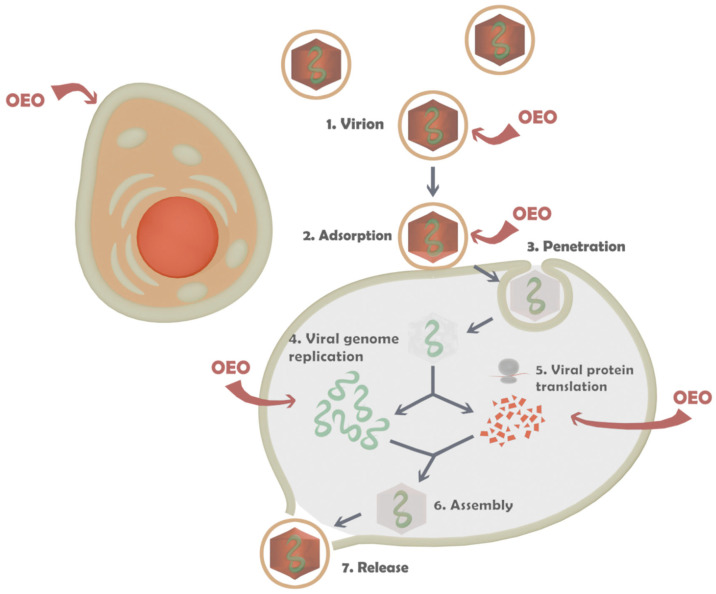
Stages of viral reproduction treated with *Origanum vulgare* ssp. *hirtum* essential oil.

To determine the antiviral activity of pure oregano oil, blank PMs, and OEO-loaded PMs, four different experimental setups were applied at different stages of viral reproduction. In the first setup (1), the effect on the replication of the three types of viruses in the respective susceptible cells was studied. In the second setup (2), the effect on the stage of viral adsorption to the host cells was monitored. The third setup (3) determined the virucidal effect of the studied products when directly affecting extracellular virions. The fourth experimental model (4) demonstrated the protective effect of the oil and the carrier when pre-treating healthy cells with the aim of protection from subsequent viral infection (Figure 7).

In the experiments focused on determining the effect of oregano oil, the micellar nanocarrier, and OEO-loaded micelles on viral replication, none of the samples showed a significant effect on any of the stages of viral replication. The inhibition was less than 50% for all three samples against the three viruses tested. Therefore, the selectivity index of the tested products could not be determined.

When studying the influence of OEO on the adsorption of HSV-1, a distinct inhibitory effect was found even at the first studied time interval (15 min) with a decrease in the viral titer Δlg = 1.75 (Table 4). A clear dependence was observed, in which the effect of the tested systems increases with increasing exposure time. At the last time interval, the decrease in the viral titer was Δlg = 2.5. The blank PEO-*b*-PCL micelles initially did not show a significant influence, but an effect was observed at 30 min, which became more pronounced at/after 45 min, characterized by a decrease in the viral titer by Δlg = 1.75. The strongest effect was observed for OEO-loaded PEO-*b*-PCL micelles, which inhibited time-dependently the adsorption of the virus with Δlg = 2.0 (at 15 min) to Δlg = 3.0 (60 min) (Table 4).

The adsorption of HCoV OC-43 was affected by OEO and PEO-*b*-PCL micelles, evident from the decrease in viral titers by Δlg = 2.0 during all monitored time intervals. The effect of OEO-loaded PMs was slightly stronger than pure substances, as the system inhibited the process by Δlg = 2.25 during the entire period of viral adsorption (Table 4).

**Figure 8:**
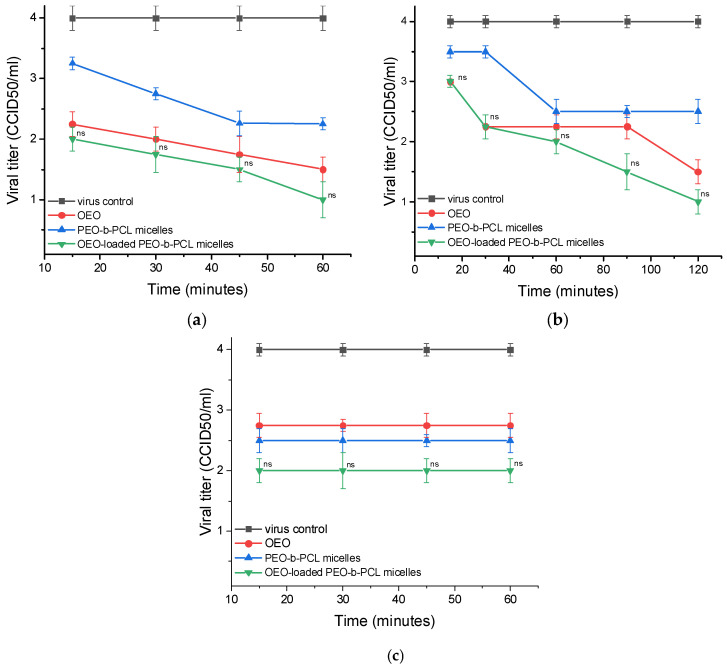
Influence on the viral adsorption stage. (**a**)—influence on the stage of adsorption of HSV-1 to MDBK cells; (**b**)—influence on the stage of adsorption of HCoV OC43 to Vero E6 cells; (**c**)—influence on the stage of adsorption of FCV to CRFK cells; ns—no significant difference (*p* > 0.05), when comparing the value of each OEO-loaded PEO-*b*-PCL micelles with the value of OEO for the corresponding time interval, Student’s *t*-test.

Concerning FCV cell adsorption, the viral titer Δlg = 2.5 upon treatment with pure OEO for 15 min and slightly increased at/after 30 min to Δlg = 2.75. Blank PEO-*b*-PCL micelles also demonstrated a significant effect with Δlg = 2.25. The effect of OEO-loaded PEO-*b*-PCL micelles was similar to that of OEO. Accordingly, a decrease in viral titer in the range from Δlg = 2.5 (15 min) to Δlg = 2.75 (60 min) was registered (Figure 8, Table 4).

The experiments evaluating the direct effect of the systems on extracellular virions revealed that the influence of pure OEO on extracellular HSV-1 virions is initially weak, but with an increase in the exposure time, it increases, and after 90 min, a notable decrease in the viral titer Δlg = 2.75 was recorded (Table 5). Up to 60 min of treatment, blank PMs showed a negligible or weak effect, which became more significant after 90 min (Δlg = 2.0). OEO-loaded PMs demonstrated the clearest effect of the three tested samples. At 15 and 30 min, the effect was weak, Δlg = 1.5, but from 60 min onwards it increased significantly from Δlg = 2.0 to Δlg = 3.5 (at 120 min) (Table 5).

In HCoV OC-43, the effect of the three tested samples is relatively weaker than that of the other two viruses. Pure OEO and blank PMs showed a negligible to weak effect. For the OEO-loaded PMs, a significant effect was detected at 90 min with Δlg = 1.75, which slightly increased to Δlg = 2.0 at 120 min of exposure (Table 5).

**Figure 9:**
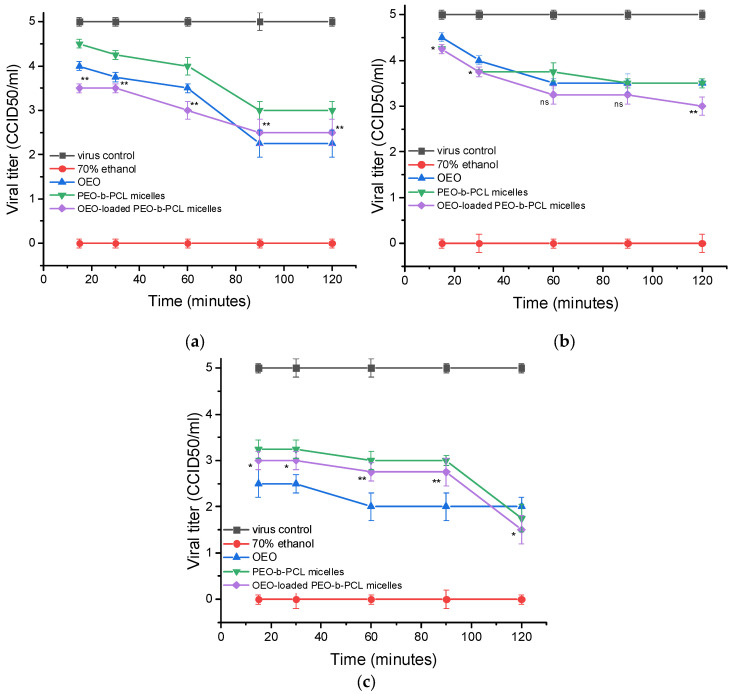
Effect on extracellular virions. (**a**)—effect on HSV-1 virions; (**b**)—effect on HCoV OC43 virions; (**c**)—effect on FCV virions; ns—no significant difference (*p* > 0.05), * *p* < 0.05, ** *p* < 0.01, when comparing the value of each OEO-loaded PEO-*b*-PCL micelles with the value of OEO for the corresponding time interval, Student’s *t*-test.

The most pronounced virucidal activity of the systems studied was observed in FCV calls. Pure OEO at the 15th min reduced the viral titer by Δlg = 2.6, and at/after 60 min, the effect increased to Δlg = 3.0. Blank PMs also demonstrated a significant effect at 15 min (Δlg = 1.75), and the effect increased to Δlg = 3.25 at 120 min. In case of OEO-loaded PMs, the effect was slightly weaker at 15 min (Δlg = 2.0) compared to that in OEO, but it increased and at 120 min became slightly more pronounced (Δlg = 3.5) than OEO. For all three viruses, 70% ethanol was used as a control, which completely inhibited the viral particles with a decrease in viral titers by Δlg = 5.0 (Figure 9, Table 5).

When determining the protective effect of the tested samples on the three cell lines studied, it was found that OEO-loaded PMs had the most pronounced effect in all three types of cells with subsequent viral infection. In MDBK cells, the pure OEO weakly protected the cells, reducing the titer of the subsequent HSV-1 infection by at most Δlg = 1.25 after 90 min of pretreatment (Table 6). The blank PMs did not show a protective effect against MDBK cells, while treatment with OEO-loaded PMs led to significant protection with a decrease in the viral titer Δlg = 2.0 during all studied treatment time intervals (Table 6).

In the experiments with Vero E6 cells, the pure OEO showed significant protection at 30 min of incubation with cells (Δlg = 1.75), and the effect was enhanced at 120 min of treatment (Δlg = 2.5). The blank PMs showed a negligible to weak effect. OEO-loaded MPs demonstrated a distinct effect at 30 min exposure (Δlg = 1.75), which increased at 60 min (Δlg = 2.0) and reached values of Δlg = 3.0 (at 120 min) (Table 6).

**Figure 10:**
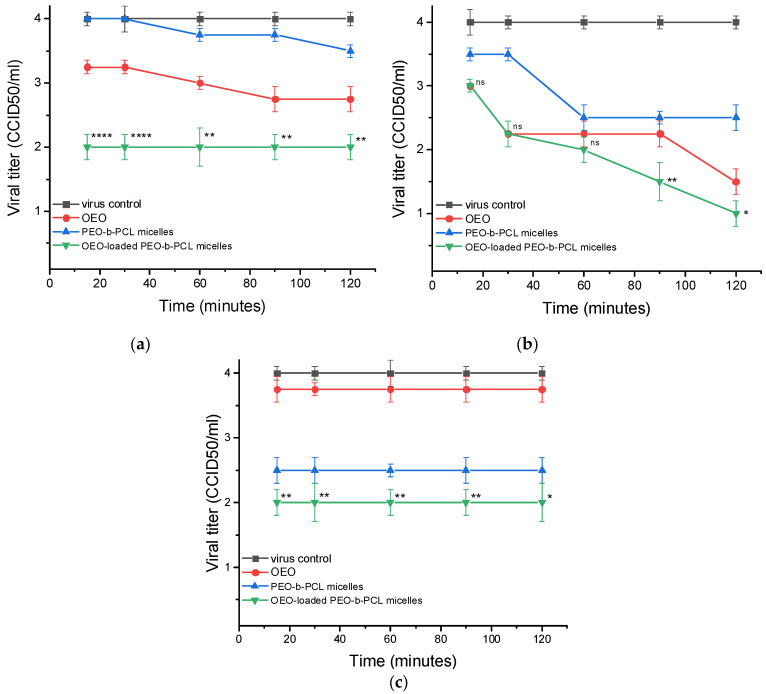
Protective effect on healthy cells. (**a**)—effect on MDBK cells; (**b**)—effect on Vero E6 cells; (**c**)—effect on CRFK cells; ns—no significant difference (*p* > 0.05), * *p* < 0.05, ** *p* < 0.01, **** *p* < 0.0001, when comparing the value of each OEO-loaded PEO-*b*-PCL micelles with the value of OEO for the corresponding time interval, Student’s *t*-test.

In CRFK cells tests, the pure OEO and blank PMs had weak protection, Δlg = 1.25 and Δlg = 1.5, respectively, while OEO-loaded PMs exhibited distinct and constant effect throughout all treatment intervals, Δlg = 2.0 (Figure 10, Table 6).

## 4. Discussion

**Figure 11:**
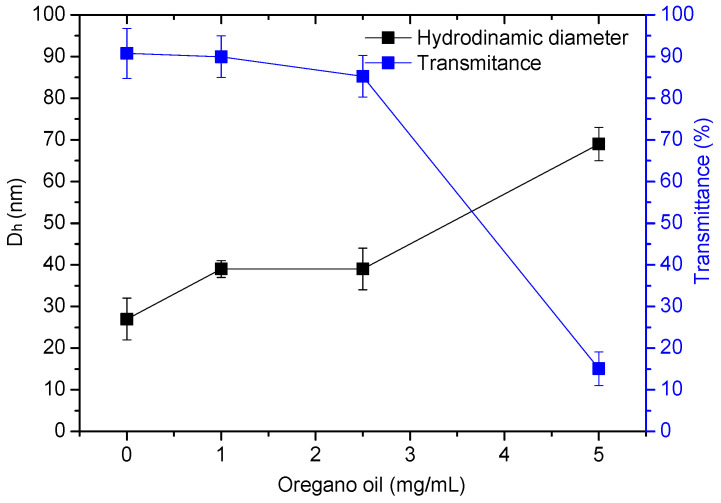
Effect of oregano oil concentration (mg/mL) on particle size (black) and transmittance (blue) of PEO-*b*-PCL micellar solutions (polymer concentration 5 mg/mL).

The strategy to encapsulate OEO into “core–shell” polymeric micelles was successfully applied to enhance the water solubility of the oil. Thus, the nanoformulation of this natural bioactive product, possessing antimicrobial, antiproliferative, antioxidant, antiparasitic, antihyperglycemic, vasoprotective, and anti-inflammatory properties [56,57,58,59] and has potential for a broad range of applications in medicine and pharmacy. The amphiphilic PEO-*b*-PCL diblock copolymer used in this study belongs to the family of biomaterials widely accepted for developing nano-sized carriers for drug delivery due to its excellent biocompatibility and biodegradability. In water, it formed structurally stable “core–shell” particles with nanoscale dimensions (D_h_ = 27 ± 5 nm), which can ensure prolonged circulation of the carrier in the bloodstream. The hydrophobic PCL core of the micelles embedded a significant amount of OEO, while the hydrophilic PEO (5000 g/mol) chains provided good colloid stability of the system. As shown, in the relatively wide range of OEO/copolymer mass ratio (from 1:5 to 1:1), PEO-*b*-PCL micelles were able to solubilize the oil and thereby notably enhance its solubility in aqueous media. Increasing the oil fraction slightly increased the D_h_ of the particles from 39 to 69 nm, which did not change the colloidal stability of the system (Figure 11). No phase separation upon storage for 7 days was observed for all samples.

The decreased transmittance, which is due to increased light scattering by the dispersed particles, suggests (besides the structural changes) the saturation of the micelle core and reaching the limit for including more OEO. Such a tendency was observed in our previous study, where the loading of Pluronic (PEO-PPO-PEO) micelles with increasing amounts of OEO resulted in high turbidity, large particle size, and reduced colloidal stability [52]. Based on the physico-chemical properties of the systems developed, OEO-loaded PEO-*b*-PCL micelles obtained at a 1:5 mass ratio were used for all biological experiments. This system is characterized by small particle size, negative zeta potential, high transparency, good colloidal stability, and contains enough OEO for further assessment of biological activity. The biphasic release profile of OEO from PCL-*b*-PEO polymeric micelles shows that at a polymer/OEO mass ratio of 5:1, only a portion of the encapsulated oil is involved in hydrophobic interactions with the PCL macromolecules, which is responsible for preventing premature diffusion of OEO into the aqueous phase. Nevertheless, these results confirm the potential of PCL-*b*-PEO nanocarriers for the delivery of oregano essential oil, providing enhanced solubility of its active compounds such as carvacrol and thymol, and minimizing their degradation.

The main problem facing the development of effective antiviral chemotherapeutic agents comes from the close relationship between the viral replicative cycle and the host cell. Therefore, targets for effective antiviral agents are those virus-specific structures and functions that determine viral replication but do not exist or are not essential for the cell, respectively, for the host organism. Viruses are obligate intracellular parasites, and in the course of their replication, the host cell provides precursors for the synthesis of viral nucleic acids, proteins, etc.; energy sources; enzymes; transfer and ribosomal RNAs; free ribosomes; and polysome complexes.

Virus-specific targets for chemotherapeutic agents are:

1. Extracellular virions. Reducing the ability of virions to infect susceptible cells is based on the ability of some chemical compounds to non-specifically inactivate virions outside the cell by denaturing viral proteins and/or causing structural changes in the lipids of the supercapsid.
2. Attachment (adsorption), in which the binding of viral structures and cellular receptors responsible for recognizing the susceptible cell by the viral particle is disrupted.
3. Virus fusion—preventing the fusion of the viral supercapsid with the cell membrane. This stops the virus from entering the cell.
4. Intracellular replicative cycle (includes transcription of viral genes, translation of viral proteins, and replication of the viral genome). Viruses induce a large number of virus-specific enzymes in the infected cell. They duplicate the action of analogous cellular enzymes but differ from them in a number of physicochemical characteristics: molecular mass, requirement for certain cations, sensitivity to inhibitors, and substrate specificity.
5. Assembly of new virus particles—if this step is affected, defective virus particles can be formed that cannot infect other cells.
6. Release from the infected cell—if this step is blocked, new cells will not be able to be infected [60].

Oregano essential oil can target different phases of the viral life cycle, such as extracellular virus, viral adsorption, internalization, or genome replication [12]. The main biological activities, including antiviral activity, of oregano oil are attributed to its main components, carvacrol and thymol. They have been shown to exhibit antiviral activity against a number of viruses [8,16,17,18], especially when pretreated. Studies by two teams have demonstrated the anti-HIV and anti-SIV effects of oregano essential oil, carvacrol, and thymol, mainly through their direct interaction with the structure of the viral capsule [19,20]. Other studies have confirmed that oregano essential oil and carvacrol only have a direct killing effect against pseudorabies virus (PRV) and limit intracellular virus proliferation, but do not inhibit PRV invasion into BHK-21 cells [21]. Oregano oil also inactivates murine norovirus within 1 h by acting directly on the viral capsid and subsequently affecting RNA [15]. Numerous studies have shown that oregano oil can directly kill lipid-encapsulated viruses [22]. Studies have shown that carvacrol and thymol block viral fusion with cells without disrupting other stages of the viral life cycle. Changes in the density of viral particles have been found, suggesting that cholesterol depletion from the HIV-1 envelope membrane occurs. Cholesterol is an important component of the viral bilayer membrane, required for viral stability and efficient fusion with the cell membrane, which results in reduced viral entry [23,24]. The dependence on cholesterol for viral entry has also been demonstrated for other enveloped viruses, such as HCV and influenza [25,26,27]. In silico studies revealed that carvacrol and thymol may show moderate activity as G-protein coupled receptor ligands, ion channel modulators, nuclear receptor ligands, protease, kinase, and enzyme inhibitors [28].

Results from other studies may help explain our results. An effect on the components of the viral envelope explains the virucidal effect observed by us and the inhibitory effect of oregano essential oil on the viral adsorption stage. The effect on cellular receptor ligands and membrane ion channels at non-toxic concentrations of the oil is the most likely explanation for the observed protective effect on healthy cells when applied immediately before viral infection.

As mentioned above, the components contained in oregano oil have an extremely low solubility in aqueous media, and to avoid this drawback, various methods are being developed for the incorporation of OEO into polymeric nanocarriers [33,34,35,36]. There have been positive results for nanoformulations in the study of antiviral activity. For example, when combining a nanogel (based on the natural polymers chitosan and albumin) with oregano oil, MDBK cells were effectively protected from betacoronavirus 1 viral infection [37]. Multivalent flexible nanogels have also been shown to exhibit broad-spectrum antiviral activity by blocking viral entry into the cell by interacting with specific protein receptors [61]. The antiviral activity of many polymers is attributed to their long chains and branches and their flexible molecular structure. Some polymers act as antiviral agents by inducing oxidative stress, producing singlet oxygen species, upon exposure to UV–visible light. Oxidative stress causes damage to macromolecules, including DNA, RNA, and proteins [62]. Polymer nanogels can attach to heparan sulfate proteoglycans on the surface of host cells and thus can prevent the entry of viral particles into host cells. These flexible nanogels, which are cross-linked hydrogel particles, serve as stable inhibitors of HSV-2 viral infections [61].

In our case, a comparative study of the antiviral activity of pure and nanoformulated OEO (encapsulated in PEO-*b*-PCL micelles) revealed a significant effect on the stage of viral adsorption, and a virucidal effect on extracellular virions was observed against all three tested viruses (HSV-1, HCoV OC-43, and FCV). We found that the effect of OEO-loaded PEO-*b*-PCL micelles is slightly more pronounced than in the free form of OEO. OEO-loaded PEO-*b*-PCL micelles also showed significant protection of healthy cells upon pretreatment before viral infection by all three tested virus types. It should be noted that the present work was limited to in vitro studies only. Forthcoming experiments are needed to confirm the observed in vitro effects in an in vivo setting, as well as to elucidate the mechanism of action of OEO. Quantification of cholesterol can also be performed to compare with the results described by other authors.

## 5. Conclusions

Encapsulating lipophilic OEO into “core–shell” PEO-*b*-PCL polymeric micelles significantly enhanced its water solubility, paving the way for a broad range of applications in medicine and pharmacy. In the range of 1:5 to 1:1 OEO/copolymer mass ratio, stable colloids were formed, with a monomodal particle size distribution and D_h_ of the particles from 39 to 69 nm. No phase separation of the systems upon storage for 7 days was observed for all samples.

A significant effect on the stage of viral adsorption and a virucidal effect on extracellular virions were observed for pure and nanoformulated OEO against the three tested viruses (HSV-1, HCoV OC-43, and FCV). The effect of OEO-loaded PMs was slightly more pronounced than the free form of oil. OEO-loaded PMs also showed significant protection of healthy cells upon pretreatment before viral infection by all three tested virus types.

Our results demonstrated that OEO-loaded PEO-*b*-PCL micelles can be successfully applied for protection against viral infections caused by both enveloped and non-enveloped viruses. The nanoformulation can also significantly limit the spread of viral particles and the stage of their attachment to susceptible cells, which can reduce the damage from viral infection and shorten the recovery period.

## Figures and Tables

**Table 1:** Hydrodynamic diameter (D_h_, nm), zeta potential (ζ-potential, mV), and transmittance (%) of blank and OEO-loaded PEO-*b*-PCL micelles at different OEO/polymer mass ratios and polymer concentration of 5 mg/mL.

Sample	Mass Ratio	D_h_ (nm)	ζ-Potential (mV)	Transmittance (%)
PEO-*b*-PCL micelles	-	27 ± 5	−8.78 ± 0.62	90.76
OEO/PEO-*b*-PCL micelles	1:5	39 ± 2	−6.41 ± 1.17	89.95
OEO/PEO-*b*-PCL micelles	1:2.5	38 ± 5	−6.51 ± 1.55	85.27
OEO/PEO-*b*-PCL micelles	1:1	69 ± 4	−8.68 ± 0.43	15.07

**Table 2:** Mean CC_50_ and photo-irritation factor (PIF) in BALB 3T3 cells.

Compounds	CC_50_ ± SD (µg/mL)		PIF
	Non-Irradiated	Irradiated	
Blank polymer micelles	159.59 ± 9.27	67.66 ± 2.91	2.4
Oregano oil	93.25 ± 2.02	90.70 ± 2.27	1.0
Oregano oil-loaded polymer micelles	105.05 ± 2.05	54.17 ± 3.22	1.9
Chlorpromazine	18.23 ± 0.68	3.17 ± 0.52	5.8

**Table 3:** In vitro assessment of cytotoxicity of pure OEO, blank- and OEO-loaded PMs.

Viral Strain	Time	Samples Concentration (µg/mL)				
		OEO	PMs	OEO-Loaded PMs	Acyclovir	Remdesivir
MDBK	CC_50_	294.6 ± 5.7 *	530.4 ± 6.3 ***	340.7 ± 5.4 ***	291.0 ± 9.4	nd
	MTC	50	100	100	nd	nd
Vero E6	CC_50_	228.3 ± 4.6 ***	500.0 ± 5.3 ***	310.0 ± 4.9 ***	nd	245.0 ± 5.6
	MTC	50	100	100	nd	nd
CRFK	CC_50_	262.4 ± 4.2	520.5 ± 6.4	324.6 ± 4.4	nd	nd
	MTC	50	100	100	nd	nd

nd—no data; * *p* < 0.05, when comparing the value of each sample with the corresponding reference substance for the given cell line; *** *p* < 0.001, when comparing the value of each sample with the corresponding reference substance for the given cell line.

**Table 4:** Influence of pure OEO, blank, and OEO-loaded PMs on the viral adsorption stage.

Viral Strain	Time	Δlg ± SD		
		OEO	PEO-*b*-PCL Micelles	OEO-Loaded PEO-*b*-PCL Micelles
HSV-1	15 min	1.75 ± 0.2	0.75 ± 0.1	2.0 ± 0.2 ^ns^
	30 min	2.0 ± 0.2	1.25 ± 0.1	2.25 ± 0.3 ^ns^
	45 min	2.25 ± 0.3	1.75 ± 0.2	2.5 ± 0.2 ^ns^
	60 min	2.5 ± 0.2	1.75 ± 0.1	3.0 ± 0.3 ^ns^
HCoV OC-43	15 min	2.0 ± 0.3	2.0 ± 0.2	2.25 ± 0.2 ^ns^
	30 min	2.0 ± 0.2	2.0 ± 0.2	2.25 ± 0.2 ^ns^
	60 min	2.0 ± 0.1	2.0 ± 0.2	2.25 ± 0.2 ^ns^
	90 min	2.0 ± 0.2	2.0 ± 0.1	2.25 ± 0.3 ^ns^
	120 min	2.0 ± 0.2	2.0 ± 0.2	2.25 ± 0.2 ^ns^
FCV	15 min	2.5 ± 0.3	2.25 ± 0.2	2.5 ± 0.2 ^ns^
	30 min	2.75 ± 0.3	2.25 ± 0.3	2.5 ± 0.3 ^ns^
	45 min	2.75 ± 0.2	2.25 ± 0.2	2.5 ± 0.2 ^ns^
	60 min	2.75 ± 0.3	2.25 ± 0.2	2.75 ± 0.3 ^ns^

^ns^—no significant difference (*p* > 0.05), when comparing the value of each OEO-loaded PEO-*b*-PCL micelles with the value of OEO for the corresponding time interval, Student’s *t*-test.

**Table 5:** Virucidal activity of pure OEO, blank, and OEO-loaded PMs.

Viral Strain	Time	Δlg ± SD			
		OEO	PEO-*b*-PCL Micelles	OEO-Loaded PEO-*b*-PCL Micelles	70% Ethanol
HSV-1	15 min	1.0 ± 0.1	0.5 ± 0.1	1.5 ± 0.1 **	5.0 ± 0.1
	30 min	1.25 ± 0.1	0.75 ± 0.1	1.5 ± 0.1 *	5.0 ± 0.1
	60 min	1.5 ± 0.1	1.0 ± 0.2	2.0 ± 0.2 *	5.0 ± 0.1
	90 min	2.75 ± 0.3	2.0 ± 0.2	3.5 ± 0.3 *	5.0 ± 0.1
	120 min	2.75 ± 0.3	2.0 ± 0.2	3.5 ± 0.3 *	5.0 ± 0.1
HCoV OC-43	15 min	0.5 ± 0.1	0.75 ± 0.1	0.75 ± 0.1 *	5.0 ± 0.1
	30 min	1.0 ± 0.1	1.25 ± 0.1	1.25 ± 0.1 *	5.0 ± 0.1
	60 min	1.5 ± 0.1	1.25 ± 0.2	1.75 ± 0.2 ^ns^	5.0 ± 0.1
	90 min	1.5 ± 0.2	1.5 ± 0.1	1.75 ± 0.2 ^ns^	5.0 ± 0.1
	120 min	1.5 ± 0.1	1.5 ± 0.1	2.0 ± 0.2 **	5.0 ± 0.1
FCV	15 min	2.5 ± 0.3	1.75 ± 0.2	2.0 ± 0.2 *	5.0 ± 0.1
	30 min	2.5 ± 0.2	1.75 ± 0.2	2.0 ± 0.2 *	5.0 ± 0.1
	60 min	3.0 ± 0.3	2.0 ± 0.2	2.25 ± 0.2 **	5.0 ± 0.1
	90 min	3.0 ± 0.3	2.0 ± 0.1	2.25 ± 0.3 **	5.0 ± 0.1
	120 min	3.0 ± 0.2	3.25 ± 0.3	3.5 ± 0.3 *	5.0 ± 0.1

^ns^—no significant difference (*p* > 0.05), * *p* < 0.05, ** *p* < 0.01, when comparing the value of each OEO-loaded PEO-*b*-PCL micelles with the value of OEO for the corresponding time interval, Student’s *t*-test.

**Table 6:** Protective effect of pure OEO, blank, and OEO-loaded PMs upon pretreatment of cells.

Viral Strain	Time	Δlg ± SD		
		OEO	PEO-*b*-PCL Micelles	OEO-Loaded PEO-*b*-PCL Micelles
HSV-1	15 min	0.75 ± 0.1	0.0	2.0 ± 0.2 ****
	30 min	0.75 ± 0.1	0.0	2.0 ± 0.2 ****
	60 min	1.0 ± 0.1	0.25 ± 0.1	2.0 ± 0.3 **
	90 min	1.25 ± 0.2	0.25 ± 0.1	2.0 ± 0.2 **
	120 min	1.25 ± 0.2	0.5 ± 0.1	2.0 ± 0.2 **
HCoV OC-43	15 min	1.0 ± 0.1	0.5 ± 0.1	1.0 ± 0.1 ^ns^
	30 min	1.75 ± 0.2	0.5 ± 0.1	1.75 ± 0.2 ^ns^
	60 min	1.75 ± 0.2	1.5 ± 0.2	2.0 ± 0.2 ^ns^
	90 min	1.75 ± 0.2	1.5 ± 0.1	2.5 ± 0.3 **
	120 min	2.5 ± 0.2	1.5 ± 0.2	3.0 ± 0.2 *
FCV	15 min	1.25 ± 0.2	1.5 ± 0.2	2.0 ± 0.2 **
	30 min	1.25 ± 0.1	1.5 ± 0.2	2.0 ± 0.3 **
	60 min	1.25 ± 0.2	1.5 ± 0.1	2.0 ± 0.2 **
	90 min	1.25 ± 0.2	1.5 ± 0.2	2.0 ± 0.2 **
	120 min	1.25 ± 0.2	1.5 ± 0.2	2.0 ± 0.3 *

^ns^—no significant difference (*p* > 0.05), * *p* < 0.05, ** *p* < 0.01, **** *p* < 0.0001, when comparing the value of each OEO-loaded PEO-*b*-PCL micelles with the value of OEO for the corresponding time interval, Student’s *t*-test.

## Data Availability

Data is available from the authors.

## Supplementary Materials

The following supporting information can be downloaded at: https://www.mdpi.com/article/10.3390/biomedicines13102417/s1: Figure S1. ^1^H-NMR spectrum of PEO_113_-*b*-PCL_29_ diblock copolymer in CDCl_3_; Figure S2. GPC chromatograms of PEO_113_-OH and PEO_113_-*b*-PCL_29_ diblock copolymer; Figure S3. Calibration curve of oregano oil in phosphate buffer pH 7; Table S1. Composition and molecular characteristics of PEO_113_-OH and PEO_113_-*b*-PCL_29_ diblock copolymer; Table S2. Calculation of the concentration of the main compounds of *Origanum vulgare* ssp. *hirtum* essential oil by GC-MS.
