# Supplementary material for: Antiviral Activity of Origanum vulgare ssp. hirtum Essential Oil-Loaded Polymeric Micelles
Source: Biomedicines. 2025 Oct 2;13(10):2417. doi: 10.3390/biomedicines13102417 (PMC12561757; doi:10.3390/biomedicines13102417)
Supplement: Supplementary file 1 [file biomedicines-13-02417-s001.zip › biomedicines-3829202-supplementary.pdf]

biomedicines

MDPI

Supplementary Materials:

Antiviral Activity of *Origanum vulgare* ssp. *hirtum* Essential Oil-Loaded Polymeric Micelles

Neli Vilhelmova-Ilieva [1], Ivan Iliev [2], Katya Kamenova [3,4], Georgy Grancharov [3], Krasimir Rusanov [4,5], Ivan Atanassov [4,5] and Petar D. Petrov [3,4,*]

1. *Synthesis and characterization of diblock copolymer*

Polymerization was carried out in an oven-dried 50 mL two-neck round-bottom flask under an inert atmosphere. In a typical run, PEG_{113}–OH (1.5 g, 0.3 mmol, 1 eq.), dried by azeotropic distillation from anhydrous toluene, ε-CL (1.2 g, 10.5 mmol, 35 eq.) and toluene (15 mL) were placed in the flask. The solution was degassed by argon flow for 20 min and catalyst $Sn(Oct)_2$ (21.5 mg, 0.053 mmol, 0.5 mol% of the monomer) was added, followed by further degassing for 20 min. The reaction mixture was stirred at 115 °C for 24 h and poured into cold isopropanol. The precipitated copolymer was collected by filtration and dried thoroughly in vacuum. Yield: 2.6 g.

^{1}H NMR (600 MHz, $CDCl_3$) δ (ppm) = 4.22 (t, 2H, -CH_2O-), 4.05 (t, 57H, -CH_2O-), 3.50-3.85 (m, 450H, -CH_2O-), 3.37 (s, 3H, CH_3-O-), 2.31 ((t, 57H, -COCH_2O-), 1.55-1.80 (m, 114H, CH_2-CH_2-CH_2-CH_2-O-), 1.33-1.46 (m, 57H, $COCH_2$CH_2).

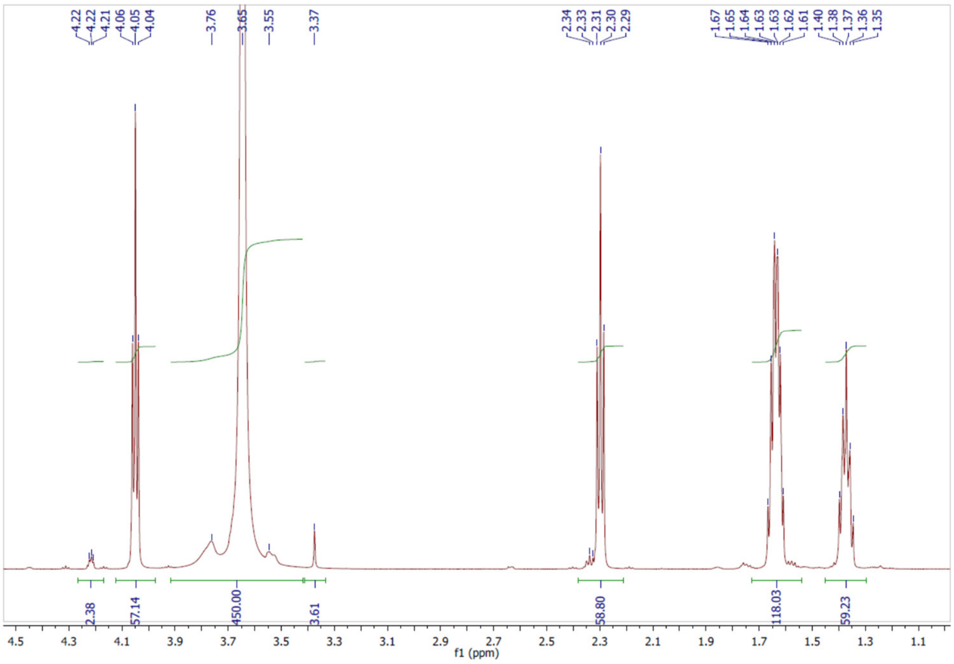

Figure S1. *^{1}H-NMR spectrum of PEO_{113}-b-PCL_{29} diblock copolymer in $CDCl_3$.*

 https://doi.org/10.3390/xxxxx

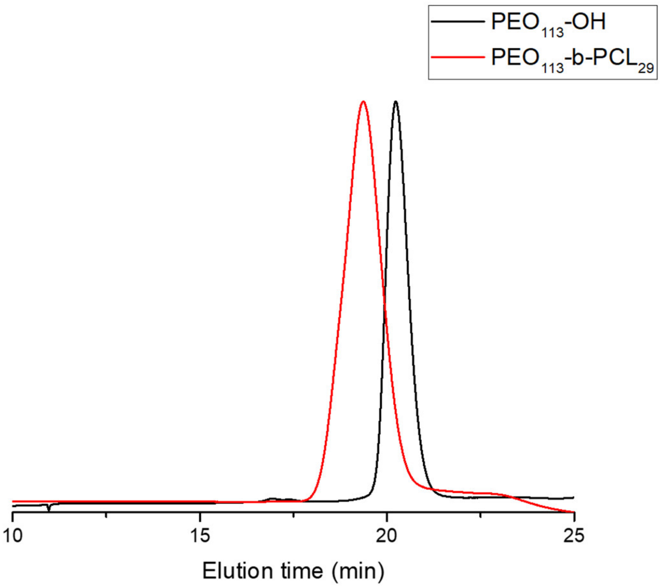

Figure S2. *GPC chromatograms of PEO_{113}-OH precursor and PEO_{113}-b-PCL_{29} diblock copolymer.*

Table S1. *Composition and molecular characteristics of PEO_{113}-OH precursor and PEO_{113}-b-PCL_{29} diblock copolymer.*

Copolymer	$M_n^{(NMR)}$ (g/mol)	$M_n^{(SEC)}$ (g/mol)	$DI^{(SEC)}$
PEO_{113}-*b*-PCL_{29}	8310	7950	1.12
PEO_{113}-OH	5000	4970	1.03

2. *GC–MS/FID Analysis of Oregano Essential Oil*

A total of 20 microliters of OEO were diluted with 0.38 mL hexane and the obtained diluted OEO was used for GC–MS/FID analysis. The analysis was performed on an Agilent 8890 GC system equipped with an Agilent 5977B mass spectrometer with a FID detector (Agilent Technologies, Santa Clara, CA, USA). The compounds were separated on an Agilent HP-INNOWax column (30 m × 0.25 mm, 0.25 µm) utilizing PEG as a stationary phase with helium 5.0 (purity 99.999 vol.%) as a carrier gas at a constant flow of 0.8 mL/min. One microliter of essential oil was injected using a split of 1:100 and the following acquisition parameters: injector temperature 250 °C. Oven program: initial temperature 65 °C, then 2 °C/min to 170 °C, hold for 0 min, then 60 °C/min to 240 °C, hold for 15 min, run time 68.667 min. The 5977B mass-selective detector was operated at a transfer line temperature of 250 °C, electron impact ionization voltage of 70 eV and quadrupole temperature of 150 °C. The FID detector was operated at 300 °C. Normal alkanes C10–C40 (Sigma-Aldrich via FOT, Sofia, Bulgaria) were used for RI calculation using AMDIS ver. 2.71 (National Institute of Standards and Technology (NIST), USA). Compound identification was carried out based on comparison of their mass spectrum and RI data with the NIST 2008 Mass spectral library (National Institute of Standards and Technology (NIST), USA) and literature data. Relative quantification of each compound expressed as percentage of the total chromatogram area was performed based on data from the FID detector.

The composition of the oil after loading into the polymer nanocarriers was determined by the same procedure.

Table S2. *Calculation of the concentration of the main compounds of Origanum vulgare ssp. hirtum essential oil by GC-MS*

	**Compound**	**RT**	**RI**	**%**
1	α-Pinene	8,472	1006,3	0,034
2	α-Thujene	8,598	1009,7	0,067
3	β-Myrcene	13,8764	1152,2	0,1
4	α-Terpinene	14,6221	1172,3	0,044
5	γ-Terpinene	18,0297	1242,5	0,312
6	3-Octanone	18,5001	1250,9	0,098
7	p-Cymene	19,4545	1267,9	0,473
8	1-Octen-3-ol	29,9472	1451,9	0,58
9	cis-Sabinene hydrate	30,7156	1464,8	1,069
10	Linalool	35,8088	1550,3	0,372
11	Caryophyllene	38,4927	1595,4	0,874
12	Terpinen-4-ol	38,92	1602,7	0,361
13	Carvacrol methyl ether	39,236	1608,4	0,417
14	trans-Dihydrocarvone	40,3269	1627,8	0,165
15	Humulene	42,6583	1669,3	0,187
16	α-Terpineol	44,3779	1700	0,133
17	endo-Borneol	44,5371	1702,8	0,976
18	β-Bisabolene	46,0123	1729,1	0,379
19	Carvone	46,3356	1734,9	0,191
20	α-Farnesene	47,3274	1752,5	0,109
21	Caryophyllene oxide	55,2654	1992,8	0,18
22	Spathulenol	57,4172	2140,6	0,075
23	Thymol	58,1816	2195,6	1,618
24	Carvacrol	58,6649	2226,8	90,306
	Total identified			99,12

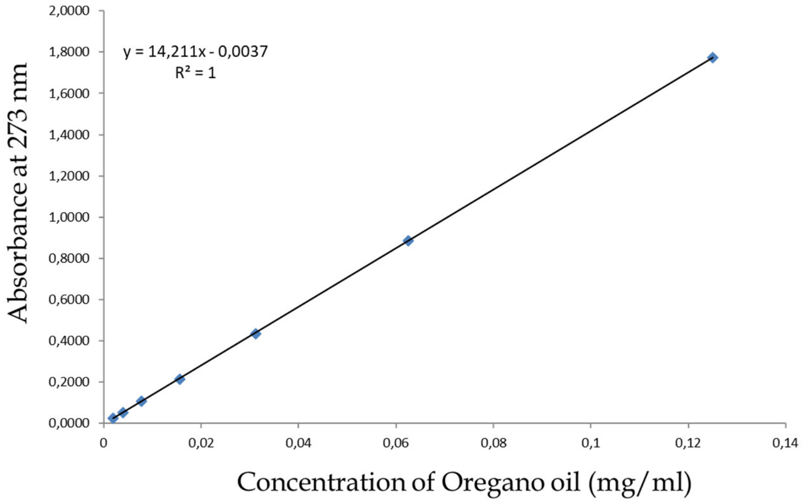

Figure S3. *Calibration curve of Oregano oil in phosphate buffer pH 7.*
